# Are inspectors’ assessments reliable? Ratings of NHS acute hospital trust services in England

**DOI:** 10.1177/1355819616669736

**Published:** 2016-10-05

**Authors:** Alan Boyd, Rachael Addicott, Ruth Robertson, Shilpa Ross, Kieran Walshe

**Affiliations:** 1 Research Fellow in Healthcare and Public Sector Management, Alliance Manchester Business School, University of Manchester, UK; 2 Head of Research, The King’s Fund, UK; 3 Research Fellow, The King’s Fund, UK; 4 Senior Researcher, The King’s Fund, UK; 5 Professor of Health Policy and Management, Alliance Manchester Business School, University of Manchester, UK

**Keywords:** Care Quality Commission, hospitals, inspections, regulation, reliability

## Abstract

The credibility of a regulator could be threatened if stakeholders perceive that assessments of performance made by its inspectors are unreliable. Yet there is little published research on the reliability of inspectors’ assessments of health care organizations’ services.

**Objectives:**

We investigated the inter-rater reliability of assessments made by inspectors inspecting acute hospitals in England during the piloting of a new regulatory model implemented by the Care Quality Commission (CQC) during 2013 and 2014. Multi-professional teams of inspectors rated service provision on a four-point scale for each of five domains: safety; effectiveness; caring; responsiveness; and leadership.

**Methods:**

In an online survey, we asked individual inspectors to assign a domain and a rating to each of 10 vignettes of service information extracted from CQC inspection reports. We used these data to simulate the ratings that might be produced by teams of inspectors. We also observed inspection teams in action, and interviewed inspectors and staff from hospitals that had been inspected.

**Results:**

Levels of agreement varied substantially from vignette to vignette. Characteristics such as professional background explained only a very small part of the variation. Overall, agreement was higher on ratings than on domains, and for groups of inspectors compared with individual inspectors. A number of potential causes of disagreement were identified, such as differences regarding the weight that should be given to contextual factors and general uncertainty about interpreting the rating and domain categories.

**Conclusion:**

Groups of inspectors produced more reliable assessments than individual inspectors, and there is evidence to support the utility of appropriate discussions between inspectors in improving reliability. The reliability of domain allocations was lower than for ratings. It is important to define categories and rating levels clearly, and to train inspectors in their use. Further research is needed to replicate these results now that the model has been fully implemented, and to understand better the impact that inspector uncertainty and disagreement may have on published CQC ratings.

## Introduction

Systems of inspection almost always involve inspectors assessing organizational performance against standards, based on information that has been gathered about the services that the organization provides. These assessments then form the basis for action by the regulator, the organization itself and various other actors. If stakeholders perceive that assessments of performance are unreliable, then the credibility of the whole system of regulation could be undermined.

One form of reliability is inter-rater reliability (sometimes termed inter-rater agreement, or reproducibility). This occurs if different inspectors arrive at the same conclusions when presented with the same evidence.

Little research has been published concerning the reliability of inspectors’ assessments of health care services. What research there is suggests that reliability varies depending on the nature of the inspection regime.^1^ The high inter-rater reliability of two accreditation survey teams simultaneously assessing a large Australian teaching hospital was attributed to consistent application of standards.^2^ Reliability of inspector assessments of Australian nursing homes has also been found to be high, in contrast with the low reliability researchers have observed in the US.^3^ A smaller number of broader standards may be more reliable than a large number of detailed standards. This is partly because inspectors can maintain a constant focus on all of the standards during an inspection in the former case. In addition, all team members can also systematically discuss whether enough data have been collected to reach a valid rating on each standard, or whether to collect additional information.

Three studies have considered assessments made by inspectors from the Dutch Healthcare Inspectorate, IGZ. A study of assessments made by nursing home inspectors found statistically significant differences in assessments between inspectors with regard to 14 out of 25 assessment criteria.^4^ Hospital inspectors, using a lightly structured regulatory instrument, demonstrated widely differing interpretations of what each assessment criterion meant and this was also the case for nursing home inspectors using a highly structured instrument.^5^ The most recent study identified various potential sources of variation in assessments of nursing home care; some concerning the instrument itself and others related to differing inspector perspectives on regulation and inspection.^6^ Agreement might be improved by prior participation in a consensus meeting and by increasing the number of inspectors.

Previous research on inspections of the organizations (National Health Service (NHS) trusts) that run public hospitals in England has highlighted wide variations in inspection processes, due in part to variations in the backgrounds, experience and skills of inspectors.^7,8^ Reviews of the Care Quality Commission (CQC), the current regulator of health and social care services in England, have identified some inconsistencies in regulatory decision-making, with some regulated organizations perceiving that inspections were overstating minor problems,^9^ and that some inspectors lacked the expertise to assess risk effectively.^10^

During 2013 and 2014, CQC piloted its new regulatory model for acute hospitals in England (Table 1),^11^ and has since rolled out variants of this model to other sectors. Inspections now emphasize expert judgement within a framework of broad standards, rather than a detailed checklist. Inspectors are, however, provided with a generic list of ‘key lines of enquiry’ and associated statistical data, which they may use to prioritize the issues they wish to investigate during the inspection visit.

**Table 1.** The new regulatory model for assessing NHS acute hospitals in England.

Domains	Rating categories	Service areas	Organization of inspections
• Safety • Effectiveness • Caring • Responsiveness • Leadership	• Inadequate • Requires improvement • Good • Outstanding	• Children and young people • Maternity and gynaecology • Urgent and emergency services • Outpatients and diagnostic imaging • Surgery • Medical care, including older people’s care • Critical care • End of life care	• Large team • Sub-teams of 3–5 inspectors rate performance for each service area with regard to each domain. Sub-team membership: ^ Led by an experienced inspector employed by CQC ^ A doctor, a nurse, and a manager with experience of the area ^ Patient advocate, trainee doctor or nurse in some sub-teams • Typically 1–2 days inspection per hospital site. Announced in advance • Investigate pertinent issues (‘key lines of enquiry’), drawing on a generic list and statistics provided by CQC • Twice daily ‘corroboration’ discussion of likely ratings – within the sub-team and across the whole team • Optional unannounced follow-up visit to gather further data

NHS: National Health Service.

Hospital inspections are conducted by multi-professional teams of inspectors, composed of permanent CQC staff plus NHS clinicians and managers, and patient advocates (“experts by experience”). Teams rate service provision across five domains (safety, effectiveness, caring, responsiveness and leadership), using a four-point scale (outstanding, good, requires improvement and inadequate). Inspectors hold twice daily corroborative discussions where they consider the ratings they are likely to give and where to focus their on-going data gathering. The multiple perspectives of different professionals should produce a rounded assessment, but might also reduce inter-rater reliability.

Our research investigated inter-rater reliability among inspectors and inspection teams, focusing particularly on team size and composition. Data were gathered during a CQC-funded external evaluation of the new CQC regulatory model as it was being piloted.

## Methods

### Data collection

We collected data via two surveys, interviews and observations. We surveyed members of the inspection teams for the 19 NHS hospital trusts inspected by CQC from January 2014 to March 2014, soon after their inspection visits. Respondents were asked to allocate a domain and rating to 10 vignettes, consisting of short edited extracts from previous CQC hospital inspection reports relating to a range of services, domains and rating levels (Table 2). We also sought comments on the allocation of domains and ratings to vignettes, on the allocation of domains and ratings during the pilot inspections, and on inspection processes. Two hundred and eighty-six individuals (response rate 65%) allocated domains and ratings to all 10 vignettes.

**Table 2.** Individual Inspector agreement on domain allocation and rating for the vignettes – Krippendorff’s alpha (*K*_a_) and percentage agreement (PA).

Vignette	Description		Domain						Rating					
		S	E	C	R	W	PA	*K*_a_ (unit)	I	RI	G	O	PA	*K*_a_
V1	Interpreting services are easily accessible	5.1%	15.4%	8.2%	70.9%	0.3%	53%	0.41	1.0%	3.7%	92.9%	2.4%	87%	–
V2	Complementary therapies are available to patients nearing the end of life to aid relaxation and symptom control	0.7%	11.6%	73.3%	14.0%	0.3%	57%	0.45	0.7%	2.4%	59.4%	37.5%	49%	–
V3	Staff left ampules of medicines in labour rooms instead of locking them away	97.6%	1.4%	0.7%	0.3%	0.0%	95%	0.94	63.7%	35.3%	1.0%	0.0%	53%	–
V4	Managers are developing a plan to address bullying following concerns reported in the national annual staff survey	2.4%	1.4%	0.3%	18.2%	77.7%	64%	0.54	1.0%	50.7%	47.6%	0.7%	48%	–
V5	The children's community nursing team cannot access local authority systems to check for safeguarding issues on discharge	69.5%	19.2%	0.3%	8.9%	3.1%	53%	0.40	53.6%	45.1%	1.0%	0.3%	49%	–
V6	Nurses undertake hourly rounds	26.4%	25.6%	26.0%	8.9%	3.1%	27%	0.07	0.0%	3.4%	87.4%	9.2%	77%	–
V7	New medication was researched so that a patient with a very complex condition could return home to die as they preferred	1.0%	16.1%	30.5%	51.7%	0.7%	38%	0.22	0.3%	1.4%	37.3%	61.0%	51%	–
V8	40% of staff are not up to date with their mandatory training	42.5%	17.5%	0.0%	2.4%	37.7%	35%	0.18	45.2%	54.4%	0.3%	0.0%	50%	–
V9	Systems ensure that medical patients remain under the care of the medical team when moved to another ward	26.7%	49.3%	2.4%	14.0%	7.5%	34%	0.16	0.0%	5.5%	91.4%	3.1%	84%	–
V10	Frail elderly patients with complex needs are given additional guidance and rehabilitation to prepare for surgery	3.1%	34.2%	26.7%	34.9%	1.0%	31%	0.12	0.3%	1.7%	75.5%	22.4%	63%	–
Overall agree ment							49%	0.35					61%	0.79
LL95%CI							38%	0.17					53%	0.41
UL95%CI							62%	0.53					71%	0.90

Key for column headings: domains: S, safe; E, effective; C, caring; R, responsive; W, well-led.

Ratings: I, Inadequate; RI, Requires Improvement; G, Good; O, Outstanding.

In a separate survey, senior managers and clinicians from the inspected trusts were asked to comment on the pilot inspection process and the accuracy of published CQC service ratings. These issues were also explored in over 60 qualitative telephone interviews of inspectors and hospital staff involved in the 18 inspections of acute hospital trusts conducted between September 2013 and December 2013. Inspections of six of these trusts were also observed, and a further three inspections were observed in June 2014, to check if processes had changed post-pilot.

### Data analysis

CQC ratings for each domain are produced through a complex process. Sub-teams of three to five inspectors each investigate a different service area. We observed that inspectors largely gathered evidence and allocated domains alone or in pairs, while ratings were discussed in corroborative sessions to arrive at group consensus. Ratings might also be discussed by the CQC staff members leading the sub-teams. Post-inspection, each sub-team leader draws on this information to write sections of the official report for their service area. Ratings and domain allocations are then reviewed, and sometimes amended, by the team leader and a National Quality Assurance Group.

We used the vignette data to model the domain allocations and ratings that individuals and groups of inspectors of various sizes and compositions might produce:

- Size: 1, 3, 4, 5
- Composition:
  ^ Any mix of staff;
  ^ A CQC sub-team leader plus any mix of non-CQC staff;
  ^ A diverse group of four, comprising a CQC sub-team leader, a senior doctor, a senior nurse or midwife, plus one manager, allied health professional, ‘expert by experience’ or junior clinician.
- Decision rules:
  ^ Majority vote. In the event of a tie, each tied option is equally likely.
  ^ CQC sub-team leader’s judgement unless outvoted by others. In the event of a tie, each tied option is equally likely.

In addition, we considered mergers of all possible combinations of domains and of adjacent rating categories, as such simplifications may improve reliability when raters are confusing some categories.

We also modelled the aggregate rating that might be produced when assessing a number of pieces of information. We allocated consecutive integer scores to rating categories and calculated the average score each inspector gave across the 10 vignettes, rounded to the nearest integer. We then determined group ratings using the majority vote decision rule above.

A simple method of analysing inter-rater agreement is to calculate the overall proportion of agreement (PA), i.e. the average pairwise percentage agreement for all possible pairings of inspectors that can be formed from the set of all inspectors^12^ PA has limitations in some circumstances, however.^13^ We therefore also calculated an index, Krippendorff’s alpha (*K*_a_),^13^ which takes account of the PA that would be expected by chance (PE).

*K*_a_ was selected because it can be applied straightforwardly to multiple raters, categorical data (domains) and ordinal data (ratings), with comparability between different datasets, provided appropriate weights are chosen.^14^ With a large number of raters, *K*_a_ produces similar results to other commonly used chance-corrected indices.

We estimated confidence intervals (CIs) for *K*_a_ by bootstrapping with 10,000 replications using our own Excel macros, calibrated against published software (the KALPHA SPSS macro^13^ and Agreestat software version 2015.4^15^). Published software lacks the functionality that our modelling required.

For each vignette, we also explored whether relationships existed between the ratings or domains allocated and variables representing: team member profession; seniority; past experience of different types of inspection (as an inspector, or being inspected); confidence in the accuracy of ratings made by the inspector’s sub-team during the most recent inspection; and the inspector’s rating of other vignettes. For vignettes where it was pertinent, we also considered the possession of expertise particularly relevant to the service area referred to in the vignette. First, we modelled inspectors’ domain allocations and ratings, investigating main effects using multinomial logistic regression and binary logistic regression respectively (avoiding model instability, with the vast majority of ratings for each vignette being in two adjacent categories). We then cross-tabulated statistically significant variables included in the model with the relevant rating or domain allocation.

In order to identify the nature of agreements and disagreements between inspection team members in the field, and potential causes and consequences, notes of observations of rating processes during inspections were summarized in a semi-structured template. Interview transcripts were coded, and a thematic analysis of the text extracts coded as forming judgements was conducted. These themes were then synthesized with themes found in survey comments.

## Results

### Statistical analysis of inter-rater agreement about the vignettes

#### Domain allocation

Overall *K*_a_ was estimated to be 0.55 for groups of five inspectors (95% CI: 0.33, 0.75); 0.21 higher than for individuals (95% CI: 0.11, 0.33) (Table 2). Overall *K*_a_ was estimated to be 0.46 for groups of three and 0.51 for groups of four. Different team compositions and different decision rules produced only very small changes in *K*_a_. Levels of agreement among individuals varied substantially, from almost perfect agreement on vignette V3 (*K*_a_ = 0.94) to little better than chance agreement for vignette V6 (*K*_a_ = 0.07).

Merging the effectiveness, caring and responsive domains for groups of five inspectors increased *K*_a_ by 0.17 to 0.72 (95% CI: 0.45, 0.93), but the increase was not statistically significant (95% CI, 0.00, 0.37). Merging the caring and responsive domains increased *K*_a_ for groups of five inspectors by 0.07 to 0.62.

Multinomial logistic regression explained only a small part of the variation in domain allocation of vignettes (the maximum Cox & Snell pseudo R-squared value obtained was 0.14) and most variables were not statistically significant. Differences of professional background were statistically significant most often (in three vignettes) (Table 3), but with no uniform pattern. For example, CQC staff were more likely than other professional groups to regard vignette V9 as being primarily about responsiveness, whereas doctors were more likely to relate it to safety.

**Table 3.** Factors affecting levels of agreement on rating and domain allocations: statistically significant variables in the logistic regressions.

Vignette	Potential sources/indicators of agreement/disagreement	
	Rating level	Domain allocation
V1	• Little variation/High agreement; no significant variables	• Little variation/High agreement; no significant variables
V2	• Ratings given to other vignettes • Experience of inspections • Seniority: rated Outstanding by 72% of junior-level inspectors, compared with 36% of more senior colleagues • Content knowledge: rated Outstanding by 54% of End of life care sub-team members, compared with 35% of members of other sub-teams	• Profession: Fewer CQC staff (65%) and patients/experts by experience (65%) allocated Caring, compared with doctors (89%) and nurses/allied health professionals (73%)
V3	• Ratings given to other vignettes • Experience of inspections • Profession: rated Inadequate by 97% of Experts by Experience, compared with 60% of other inspectors	• Little variation/High agreement; no significant variables
V4	• Ratings given to other vignettes	• No significant variables
V5	• Experience of inspections • Seniority: rated Inadequate by 73% of junior level inspectors and Experts by Experience, compared with 50% of other inspectors • Profession: rated Requires Improvement by 60% of CQC inspectors, compared with 39% of other inspectors	• No significant variables
V6	• Profession: rated Outstanding by 30% of doctors, compared with 3% of other inspectors	• Perceived accuracy of ratings • Ratings given across all vignettes
V7	• Ratings given to other vignettes • Profession: rated Outstanding by 74% of nurses, compared with 57% of other inspectors	• No significant variables
V8	• No significant variables	• Profession: More CQC staff (28%) allocated Effectiveness, compared with nurses/allied health professionals (9%), doctors (11%) and patients/experts by experience (15%)
V9	• Little variation/High agreement; no significant variables	• Profession: Safety was the most popular domain allocation among doctors (48%); Effectiveness the most popular among patients/experts by experience (65%), CQC staff (58%) and nurses/allied health professionals (49%)
V10	• Ratings given to other vignettes	• Experience of inspections: Allocated Responsiveness by 28% of inexperienced inspectors, rising to 63% for the most experienced inspectors

#### Rating

For any given vignette, almost all ratings were concentrated in one or two adjacent rating categories. Overall *K*_a_ for groups of five inspectors was estimated to be 0.85 (95% CI: 0.57, 0.95); 0.06 higher than for individuals (95% CI: 0.02, 0.16). *K*_a_ for ratings was typically about 0.30 higher than *K*_a_ for domains across the different group configurations, and this was statistically significant for all but groups of five. Levels of agreement varied substantially from vignette to vignette. For example, 93% of respondents rated vignette V1 ‘Good’, whereas vignette V4 was rated ‘Requires Improvement’ by 51% and ‘Good’ by 48%.

Reducing the number of rating categories by merging the ‘Good’ and ‘Outstanding’ categories produced the greatest increase (0.05) in *K*_a_, but this was not statistically significant.

We could not calculate *K*_a_ for aggregated ratings. PA was 0.10 higher for aggregated ratings across the 10 vignettes than for ratings of individual vignettes, but this increase was not statistically significant (95% CI: −0.16, 0.36). Likewise, PA for aggregated ratings increased with group size (by 0.12 for groups of five; by 0.09 for groups of three), but these increases were not statistically significant.

Binary logistic regression explained only a small part of the variation in ratings of vignettes (the maximum Cox & Snell pseudo R-squared value obtained was 0.30). Of the variables considered, ratings given to other vignettes were most frequently significant (in five vignettes), followed by previous experience of different types of inspection (in three vignettes) and various aspects of profession or seniority (Table 3). Implications for rating and domain allocation varied. Vignette V2 illustrates this well:

- Greater experience of different types of inspection was associated with greater agreement in rating the vignette as ‘Good’ rather than ‘Outstanding’.
- End of Life sub-team members, who might have particular expertise in relation to this vignette, were, less likely however, than other sub-team members to rate the vignette as ‘Good’. Agreement among End of Life sub-team members was low.
- Junior clinicians had relatively high agreement among themselves, but low agreement with other profession/seniority groups.

### Qualitative analysis

#### Domain allocation

Difficulty in determining domains during inspections was a common theme in survey comments, interviews and observations. For example:

‘The domains were the issue that I found that lacked clarity. … during the group feedback sessions it was clear that lots of others were equally unsure’ (Board level nurse, inspection team member)

‘[the ratings] did change quite dramatically when we finally pulled the report together. But the reason why it changed was that debate over which domain does this fit in … Is this safety? Is this responsive? Is this caring? And that's a greying area … Which domain does [it] fit under? Have we read this description right?’ (CQC inspection team leader)

Some of this difficulty appeared to be intrinsic to the measurement categories themselves, while other issues related to their practical implementation. One particular element of service provision can have an impact on several domains. For example:

‘You can't have a well led organisation that has got poor scores across the rest of the domains. That would make no sense at all, it would look absurd.’ (Medical Director, inspection team chair)

‘Lack of translation [services for non-English speaking patients] makes assessment and care less safe, yet provision [of translation services] is responsive to the needs of the patient and ensures more effective assessment’ (Expert by experience, inspection team member)

Difficulties with the leadership domain were most prominent. Some inspectors considered it hard to distinguish from other domains, and were unclear about what level of leadership they should be assessing:

‘The hardest one to rate I think was well led … there’s actually very little quantitative data … It’s based particularly on what one hears. It’s point in time. And also, … Are we talking about leadership at a user level, a service level, or a systems level?’ (Medical Director, inspection team member)

#### Rating

Determining ratings could also be problematic. Some inspectors found the ’Requires Improvement’ category contradictory, as every service should always be striving to improve. Others suggested that ‘Requires Improvement’ and ‘Good’ could span a wide range of performance levels, partly because they might be less open to challenge than the end categories of the scale. Difficulties in distinguishing between ‘Good’ and ‘Outstanding’ were highlighted the most in our data.

‘I think the real problem we got into was what is the difference between good and outstanding?… the trouble is that within any trust you will find things that aren’t right … and the question is what does that mean? … [inspectors] are very focused on finding the things that aren’t right … And it’s quite hard for them to balance that against the really good things.’ (Consultant, inspection team chair)

Many survey respondents stressed the importance of the context when assessing pieces of evidence, but there were differences regarding the weight that should be given to some contextual factors. One example was the situation of recently appointed leaders tackling deep-seated problems, but who needed more time fully to address them. CQC policy is for ratings to reflect current quality rather than recent action or trends, but some inspectors felt that this might be counterproductive, and assessed accordingly:

‘Where there is a genuine feel and evidence that a service is moving towards improvement, then a rating that is borderline for “Inadequate”, for example, may move to “Requires Improvement”.’ (Professional advisor, inspection team member)

We also observed ratings being shaped by inspectors’ prior experiences and backgrounds. Some interviewees suggested that experienced clinicians might be inclined to rate higher than other inspectors because they appreciated the potential adverse impacts of low ratings on staff.

‘the people who’d … reviewed [the department] said they thought it was good. Now X said … how can you possibly call it good, they’ve had three never events in the past six months? Y … said … but what they’ve put into place … to stop any further never events are the best I’ve ever seen and they’re to be commended on it … I guess that’s the difference between somebody who is a junior doctor … and two senior guys who know what will happen if you flap somebody down when they’ve been trying their best.’ (Consultant, inspection team member)

Highly subjective definitions of terms like ‘Good’ and ‘Outstanding’ were often cited in discussions, such as defining an ‘Outstanding’ service as one you would be willing to travel 100 miles to receive, or for a member of your family to receive. Some interviewees thought that services should be assessed against absolute standards, but that appropriate criteria had not been provided. Assessments would thus likely be implicit, and relative to the performance of other NHS hospitals, of which inspectors had varying amounts of experience.

‘It's not clear how people determine how effective the service is or how strong the leadership is … if I've been the host organisation and video recorded what was going on then I'd be very concerned about it.’ (Clinical Director, inspection team member)

‘If you’ve got inspectors that come from one kind of hospital, one kind of environment, and yours is very different, you need to be careful that they don’t make judgements based on their personal experience which are really not relevant, or fair, or correctly contextualised, and we found a lot of that, and I think other people did too.’ (Chief Executive, inspected hospital)

Inspection team members commonly experienced consensus about ratings during inspections. Some were also very aware of differences between the assessments of individual inspectors, but believed that group-based decision making during inspections could resolve this. Interviewees gave examples of team members productively challenging others about their assessments. The extent of such challenge was, however, variable, due to factors such as the amount of time available, and individual attitudes and confidence.

‘[rating] actually worked. People did agree, with one exception across risk, but that was across all 40. So 1 out of 40 they disagreed with. Was actually amazing.’ (CQC inspection team leader)

‘This is where team work and corroboration are important to debate and agree an outcome as each individual team member has differing standards and expectations dependent upon experience and role.’ (Matron, inspection team member)

## Discussion

Our data indicate that during the pilot phase, individual inspectors may have assessed the same piece of information differently with regard to CQC domain and rating categories, but that groups of inspectors are likely to produce more reliable judgements. There is good evidence that domain allocation may be problematic, and more challenging than rating. We found no evidence that group composition or decision-making rules have any substantial impact on reliability. Most mergers of domain or rating categories would also appear unlikely to increase reliability.

Reasons for disagreement vary depending on the nature of the information being considered, but there are indications that some individuals may tend to err predominantly either on the high side or the low side when rating; that prior experience, particularly of different types of inspection, may sometimes affect ratings; and that profession may sometimes influence domain allocation. Such factors account for only a small proportion of the variance, however, and our qualitative data suggest that there may have been general uncertainty about interpreting the rating and domain categories during the pilot.

### Implications for policy and practice

The uncertainty among inspectors underscores the importance of clearly and fully defining categories, and of providing training for inspectors about making judgements. Both can increase reliability, with training being particularly likely to be valuable when the categories are highly subjective.^16^ Our data suggest that experience of doing inspections alone cannot be relied upon to increase agreement levels, as greater experience produced more disagreement in some instances.

The number of inspectors involved in decision-making appears to make a major contribution to the reliability of judgements, particularly with regard to domain allocation. Large teams are expensive, however, and discussion involving more individuals requires more time. We observed some problems with corroborative sessions during the pilot, including instances of insufficient time devoted to synthesis of information and discussion of assessments, different views becoming more polarized rather than an accommodation being found, and uneven involvement of team members in discussions. More training, guidance and time are indicated. According to our observations, corroborative discussions focused on ratings rather than on domain allocation, so a change of emphasis here would also likely aid reliability.

Having a mix of different professions in inspection teams does not appear to affect reliability, so policy on team composition should be determined on the basis of other considerations. For example, validity might be increased if corroborative discussions enable different perspectives to be heard and taken account of.

What level of agreement is sufficient depends on the importance of the judgements. Low CQC ratings can have far-reaching consequences including additional regulatory activities, replacement of the top management team, damage to staff morale and loss of reputation. If a low-performing service incorrectly receives a ‘Good’ rating, then poor care might continue, rather than improvements being made. A typical hospital inspection produces 40 separate ratings, which are aggregated into higher level ratings. Thus even if individual ratings have very high reliability, there may be scope for one or two ratings to be regarded as questionable. It is, therefore, important not only to seek high reliability but also to have an aggregation algorithm that is not sensitive to changes in a small number of ratings. It is arguable that this is not currently the case.

### Limitations and suggestions for further research

Our sample of vignettes is relatively small, so we cannot give very precise estimates of reliability levels, and the impact of factors such as team size. We would, therefore, suggest conducting larger scale research into aspects whose impact is potentially high but uncertain, such as merging the effectiveness, caring and responsiveness domains. Simplifying the judgement task by merging domains might not only improve reliability, but also help streamline the inspection process. Separate domains have other advantages, such as providing a focus on important aspects of quality (the CQC has been able to highlight safety issues nationally, for example^17^), but such analyses are of dubious value if domains cannot be reliably distinguished by inspectors.

It is difficult to assess the implications of our findings for published CQC ratings. The vignettes were distinct, relatively abstract, context-free pieces of information, assessed by individuals in isolation. By contrast, CQC ratings are based on a large number of pieces of information, assessed by a team of inspectors as part of an intensive inspection process lasting a number of days, and subject to change post-inspection during report writing and national quality assurance processes. Furthermore, CQC was experimenting with some aspects of the model during the period when our data were collected. Post-pilot reliability levels may be different, notwithstanding our efforts to check for changes through subsequent observation, and our analysis of the impact of inspectors’ experience on reliability. Our research focuses on the interpretations that inspectors make of the information that they have, and not on the adequacy of data collection processes during inspections. In inspections, uneven data collection processes may be an additional source of variation. Even with large inspection teams, not all parts of large service areas are inspected and this will also tend to increase variability.

Further research could enable the reliability of CQC ratings to be assessed. Such research might use a larger sample of vignettes, mapped to published CQC ratings and guidance on rating level thresholds, coupled with an investigation of data collection processes and post-inspection judgement processes.

## Conclusion

Groups of inspectors produced more reliable assessments than individual inspectors, and there is evidence to support the utility of appropriate discussions between inspectors in improving reliability. The reliability of domain allocations was lower than for ratings. Inspectors were uncertain during the pilots about interpreting the rating and domain categories, emphasizing the importance of defining categories and rating levels clearly, and of providing training. This reinforces findings from previously published studies in different settings.

Further research is needed to replicate these results now that the model has been fully implemented with updated training and guidance; to delineate more clearly where inspectors are uncertain; and to better understand the impact that inspectors’ uncertainty and disagreement may have on published CQC ratings, taking account of post-inspection judgement processes. There may also be merit in conducting further research into the utility of merging some domain categories, and to inform the development of practices to support inspection teams to discuss and reflect on the assessments they are making.

## Authors’ note

Four of the authors are currently conducting further research into the Care Quality Commission which requires the cooperation of Commission staff.
